# Exercise ameliorates endoplasmic reticulum stress-mediated vascular dysfunction in mesenteric arteries in atherosclerosis

**DOI:** 10.1038/s41598-018-26188-9

**Published:** 2018-05-21

**Authors:** Junyoung Hong, Kwangchan Kim, Eunkyung Park, Jonghae Lee, Melissa M. Markofski, Sean P. Marrelli, Yoonjung Park

**Affiliations:** 1 Laboratory of Integrated Physiology, Department of Health and Human Performance, University of Houston, Houston, TX 77204 USA; 2 Department of Neurology, McGovern Medical School at UT Health, Houston, TX 77030 USA

## Abstract

Endoplasmic reticulum (ER) stress is closely associated with atherosclerosis, but the effects of exercise on ER stress-mediated endothelial dysfunction in atherosclerosis is not yet fully understood. We assessed endothelium-dependent vasodilation in isolated mesenteric arteries from wild type (WT), WT with exercise (WT-EX), ApoE knockout (ApoE KO), and ApoE KO mice with exercise (ApoE KO-EX). Vasodilation to acetylcholine (ACh) was elicited in the presence of inhibitors of ER stress, eNOS, caspase-1, and UCP-2 (Tudca, L-NAME, AC-YVARD-cmk, and Genipin, respectively) and the ER stress inducer (Tunicamycin). Immunofluorescence was used to visualize the expression of CHOP, as an indicator of ER stress, in superior mesenteric arteries (SMA). Dilation to ACh was attenuated in ApoE KO but was improved in ApoE KO-EX. Incubation of Tudca and AC-YVARD-cmk improved ACh-induced vasodilation in ApoE KO. L-NAME, tunicamycin, and Genipin attenuated vasodilation in WT, WT-EX and ApoE KO-EX, but not in ApoE KO. Exercise training reversed the increase in CHOP expression in the endothelium of SMA of ApoE KO mice. We conclude that ER stress plays a significant role in endothelial dysfunction of resistance arteries in atherosclerosis and that exercise attenuates ER stress and regulates its critical downstream signaling pathways including eNOS, UCP-2 and caspase-1.

## Introduction

The endoplasmic reticulum (ER) is an organelle that modulates various physiological processes including protein folding, calcium homeostasis, and lipid biosynthesis for maintaining cellular homeostasis. Multiple pathologic factors, such as hyperlipidemia, oxidative stress, and calcium imbalance trigger prolonged disturbances or perturbations on ER homeostasis, leading to the unfolded protein response (UPR) pathway in the ER lumen which is known as ER stress. In prolonged ER stress, the three arms of UPR (PKR-like endoplasmic reticulum kinase (PERK), inositol-requiring enzyme 1 (IRE1), and activating transcription factor 6 (ATF6)) are activated together by the dissociation of ER chaperone BiP (GRP78) from the three sensors to restore cellular homeostasis or to activate the inflammatory pathways and apoptotic cell death^1^. ER stress is closely linked to atherosclerosis^2^; studies have reported that ER stress markers including GRP78, CHOP (CCAAT-enhancer-binding protein homologous protein), p-IRE-1, XBP-1 (X-box binding protein-1), ATF-6, p-PERK, and p-elf2 (phosphorylated-eukaryotic initiation factor), were elevated in atherosclerotic aorta in ApoE KO (apolipoprotein E knock-out) mice^3,4^.

The endothelium is recognized as a crucial site for maintaining vascular homeostasis and regulating vascular reactivity, and endothelial dysfunction is recognized as the first step toward atherosclerosis^5^. It is known that ER stress is associated with endothelial dysfunction by reduced endothelial nitric oxide synthase (eNOS) signaling^6^ and endothelium-dependent vasodilation^7^, but a comprehensive understanding of how ER stress induces endothelial dysfunction in atherosclerosis, particularly through inflammation and oxidative stress, is currently lacking. Caspase-1, which is known as the precursor of the inflammatory cytokines interleukin-1β (IL-1β) and IL-18^8,9^ is highly expressed in human atherosclerotic plaque^10^, plays a critical role in endothelial dysfunction in atherosclerosis via ER stress^9,11^. As mentioned, oxidative stress is a major risk factor for inducing ER stress and endothelial dysfunction^12^. Uncoupling protein-2 (UCP-2), which regulates mitochondrial ROS generation, may prevent a process of atherosclerosis and mitigate endothelial dysfunction with an increase in NO production in atherosclerosis and metabolic disorder^13,14^. Collectively, the ER stress-associated risk factors, inflammation and oxidative stress, causing endothelial dysfunction suggest that ER stress could be an important potential therapeutic target for vascular diseases.

It is well established that exercise has beneficial effects on endothelial function in atherosclerosis. Aerobic exercise training reversed the endothelial dysfunction observed in the aortas of LDL receptor knockout mice by increased NO bioavailability, enhanced p-eNOS (phosphorylated eNOS) expression, and down-regulation of NADPH (nicotinamide adenine dinucleotide phosphate) oxidase-derived superoxide production^15^. Furthermore, the suppression of ER stress, PERK, IRElα, and ATF6 by treadmill exercise, which also ameliorated endothelial dysfunction in diabetic aortas and mesenteric arteries, suggests that exercise improves endothelial dysfunction induced by ER stress^16^. Furthermore, regular exercise has been shown to reduce caspase-1, IL-1β, and IL-18 expression in ovariectomized rat hippocampus^17^, and increase UCP-2 expression in aged rat aorta^18^. However, the direct effects of exercise on ER stress-mediated endothelial dysfunction and on ER stress-associated caspase-1 and UCP-2 signaling in atherosclerosis merit further examination. Thus, the main objective of this study is to investigate the effects of exercise training on ER stress-mediated endothelial dysfunction in atherosclerosis and the underlying mechanisms. Our study focuses on endothelial function of mesenteric arteries to determine whether atherosclerosis impairs endothelial function in resistance vessels which are central in blood pressure regulation.

## Results

### Animal and vascular characteristics

Body weight was measured at 7–8 wks of age and after 15–16 wks of exercise training (24–25 wks of age). Although body weight at 7–8 wks of age was not different among the groups, it significantly increased in ApoE KO and ApoE KO-EX (*p* < 0.05) compared with WT and WT-EX at 24–25 wks (Table 1). This result demonstrates that ApoE gene knockout fed western diet (WD) increased the body weight regardless of the exercise training. Heart weight and heart/body weight ratio were evaluated at 24–25 wks of age and they were significantly higher in ApoE KO (*p* < 0.05) compared with all other groups. The result suggests that atherosclerosis may induce cardiac hypertrophy, and that exercise training prevented it (Table 1). Maximal intraluminal diameters of mesenteric arteries were not significantly different in WT and atherosclerotic ApoE KO vessels with or without exercise training (Table 2). No significant difference was observed in initial diameter and percentage constriction tone of mesenteric arteries in all groups (Table 2).

**Table 1:** Animal characteristics.

		Sedentary		Exercise	
		WT	ApoE KO	WT-EX	ApoE KO-EX
N		9	10	9	8
Body Weight (g)	Pre	23.52 ± 0.62	24.92 ± 1.13	24.17 ± 0.90	26.03 ± 0.59
	Post	31.32 ± 0.71	35.37 ± 1.26^*†^	29.62 ± 0.65	35.94 ± 1.37^*†^
Heart weight (mg)		150.41 ± 6.73	191.41 ± 4.69^*†^	142.83 ± 3.98	155.64 ± 5.85^#^
Heart weight/Body weight (mg/g)		4.81 ± 0.19	5.45 ± 0.18^*†^	4.84 ± 0.19	4.32 ± 0.18^#^

Body weight was significantly increased in ApoE KO and ApoE KO-EX compared with WT and WT-EX. Heart weight and heart/body weight were significantly elevated in ApoE KO mice compared with the other groups. Values are means ± SEM. ^*^p < 0.05 vs. WT, ^†^*p* < 0.05 vs^.^ WT-EX, ^#^*p* < 0.05 vs. ApoE KO.

**Table 2:** Vessel characteristics.

	Sedentary		Exercise	
	WT	ApoE KO	WT-EX	ApoE KO-EX
N	7	10	8	8
Mesentery arteries constriction tone, %	32.14 ± 6.08	33.70 ± 4.57	26.88 ± 3.15	27.38 ± 2.06
Mesentery arteries initial lumen diameter, µm	157.57 ± 19.38	138.30 ± 7.82	149.13 ± 6.23	158.13 ± 8.62
Mesentery arteries maximal lumen diameter, µm	237.43 ± 17.1	213.10 ± 10.27	205.63 ± 11.80	218.38 ± 10.33

Exercise training had no effect on the constriction tone, initial lumen, and maximal lumen of mesenteric arteries.

### Exercise training decreases atherosclerotic lesion in aorta of ApoE KO mice

**Figure 1:**
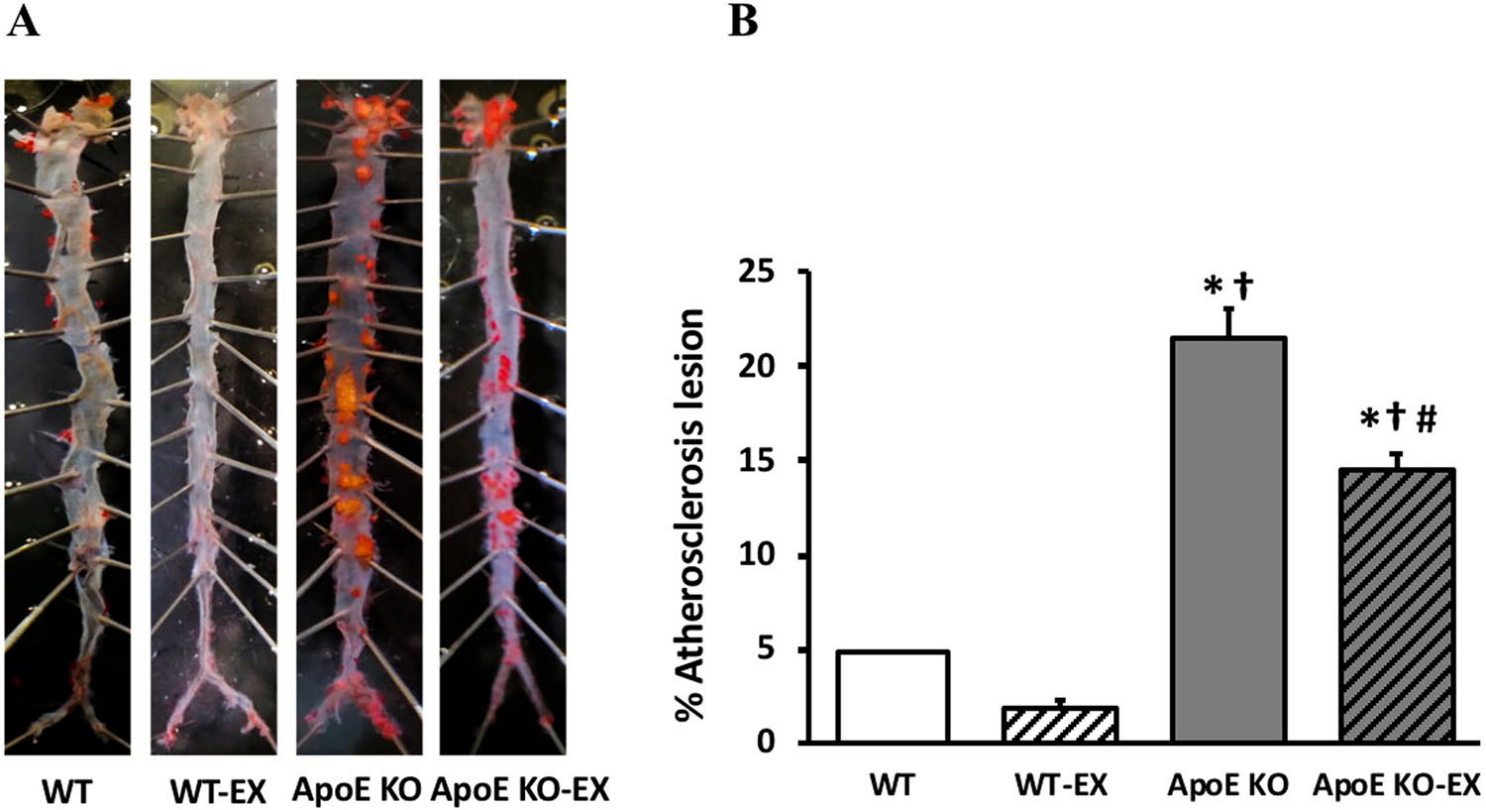
Atherosclerosis in aorta. (**A**) Atherosclerosis lesions development in aorta from four groups of mice. (**B**) Atherosclerosis lesion was significantly higher in ApoE KO (n = 3) compared with WT (n = 2), but was significantly reduced in ApoE KO-EX compared with ApoE KO. Values are means ± SEM. ^*^p < 0.05 vs. WT, ^†^*p* < 0.05 vs. WT-EX, ^#^*p* < 0.05 vs. ApoE KO.

Atherosclerotic lesion was evaluated to demonstrate the effectiveness of ApoE gene knockout fed with WD and exercise training on the development of atherosclerosis in aorta. The accumulation of atherosclerotic lesion in aortic roots was significantly higher in ApoE KO compared with WT (*p* < 0.05), but was significantly reduced by exercise training in ApoE KO-EX (*p* < 0.05; Fig. 1A, B). It tended to be lower in WT-EX mice compared to WT mice, but it was not statistically different between the groups (p = 0.157; Fig. 1A, B).

### Exercise training ameliorates endothelial dysfunction in mesenteric arteries of ApoE KO mice

**Figure 2:**
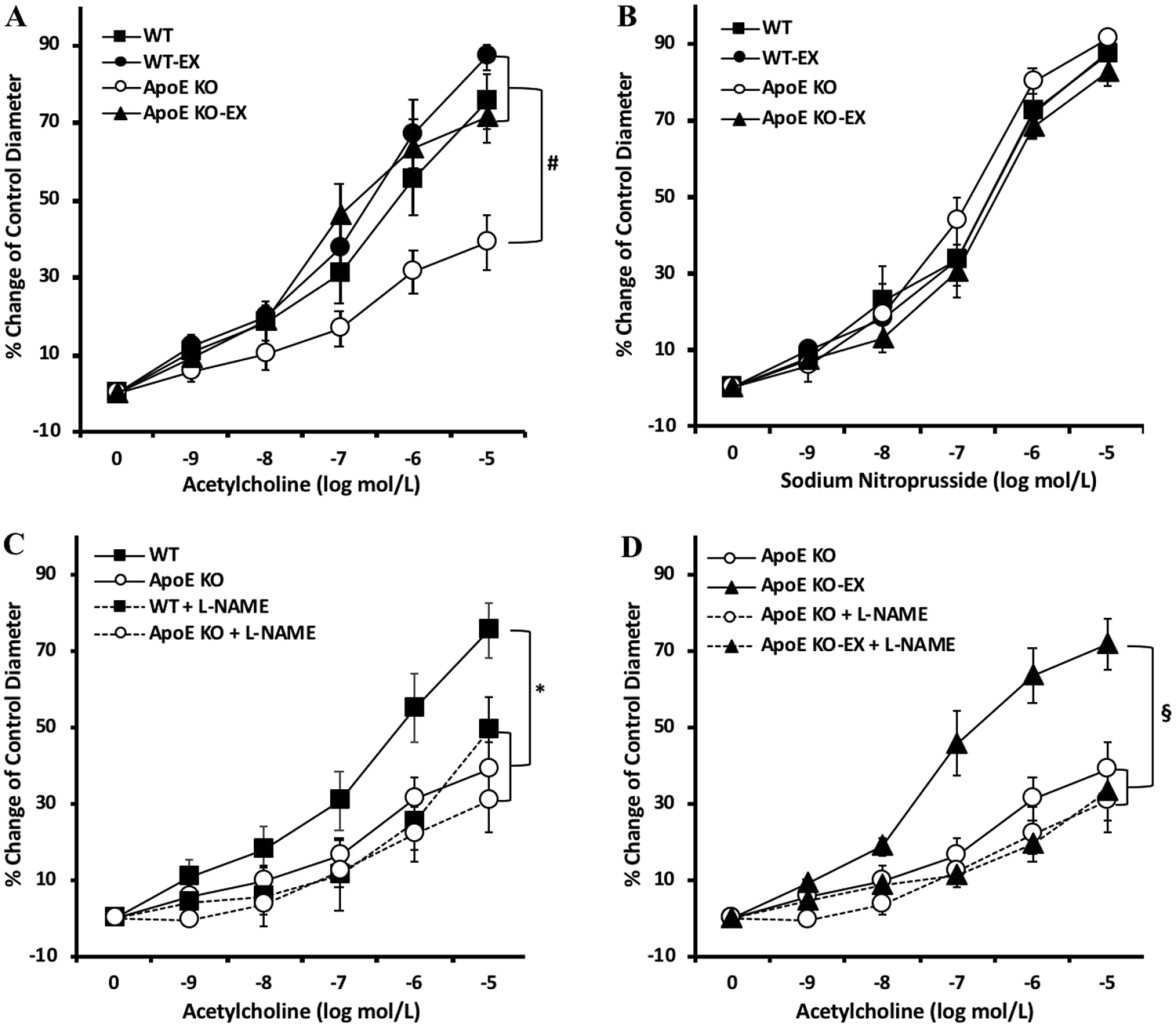
Effect of exercise training on endothelial dysfunction in mesenteric. Arteries of ApoE KO mice. (**A**) Isolated mesenteric arteries from WT (n = 7), WT-EX (n = 8), ApoE KO (n = 10), and ApoE KO-EX (n = 8) measured the response of ACh in a dose-dependent manner. ACh-induced vasodilation was significantly attenuated in the mesenteric arteries of ApoE KO mice compared with WT mice. However, exercise ameliorated the ACh-induced endothelial dysfunction in ApoE KO. (**B**) SNP-induced, the endothelium-independent vasodilation, showed no difference among the groups (WT + SNP (n = 9), WT-EX + SNP (n = 9), ApoE KO + SNP (n = 9), ApoE KO-EX (n = 8)). (**C** and **D**) Incubation of L-NAME, the eNOS inhibitor, significantly abolished endothelium-dependent vasodilation in WT and ApoE KO-EX; WT + L-NAME (n = 7), ApoE KO + L-NAME (n = 8), and ApoE KO-EX (n = 8). Values are means ± SEM. ^*^p < 0.05 vs. WT, ^#^*p* < 0.05 vs. ApoE KO, ^§^*p* < 0.05 vs. ApoE KO-EX.

To evaluate the beneficial effects of exercise training on endothelium-dependent vasodilation in atherosclerosis, ACh-induced vasodilation was measured in the isolated mesenteric arteries. ACh-induced vasodilation was significantly attenuated in mesenteric arteries of ApoE KO (*p* < 0.05) compared with WT mice, but exercise training markedly enhanced ACh-induced vasodilation in ApoE KO mice (Fig. 2A). These findings suggest that atherosclerosis impairs endothelial function in mesenteric arteries, and that exercise training ameliorates the impairment. However, vasodilation in response to endothelium-independent vasodilator (SNP; NO donor), showed no difference in the isolated mesenteric arteries among all groups (Fig. 2B). These results suggest that the alterations of vascular function by atherosclerosis and exercise training are endothelium dependent.

In addition, ACh-induced vasodilation was measured in the isolated mesenteric arteries in the presence of NOS inhibitor (L-NAME), to examine whether the NO contribution of the vasodilatory response is altered in mesenteric arteries of ApoE KO mice. Incubation with L-NAME significantly decreased ACh-induced vasodilation in the WT and ApoE KO-EX mice (*p* < 0.05), but not in ApoE KO mice (Fig. 2C, D). These results suggest that the atherosclerosis-induced endothelial dysfunction in ApoE KO and the improved endothelial function in ApoE KO mice by exercise training are a NO-dependent mechanism in mesenteric arteries.

### Exercise training alleviates ER stress-mediated endothelial dysfunction in mesenteric arteries of ApoE KO mice

**Figure 3:**
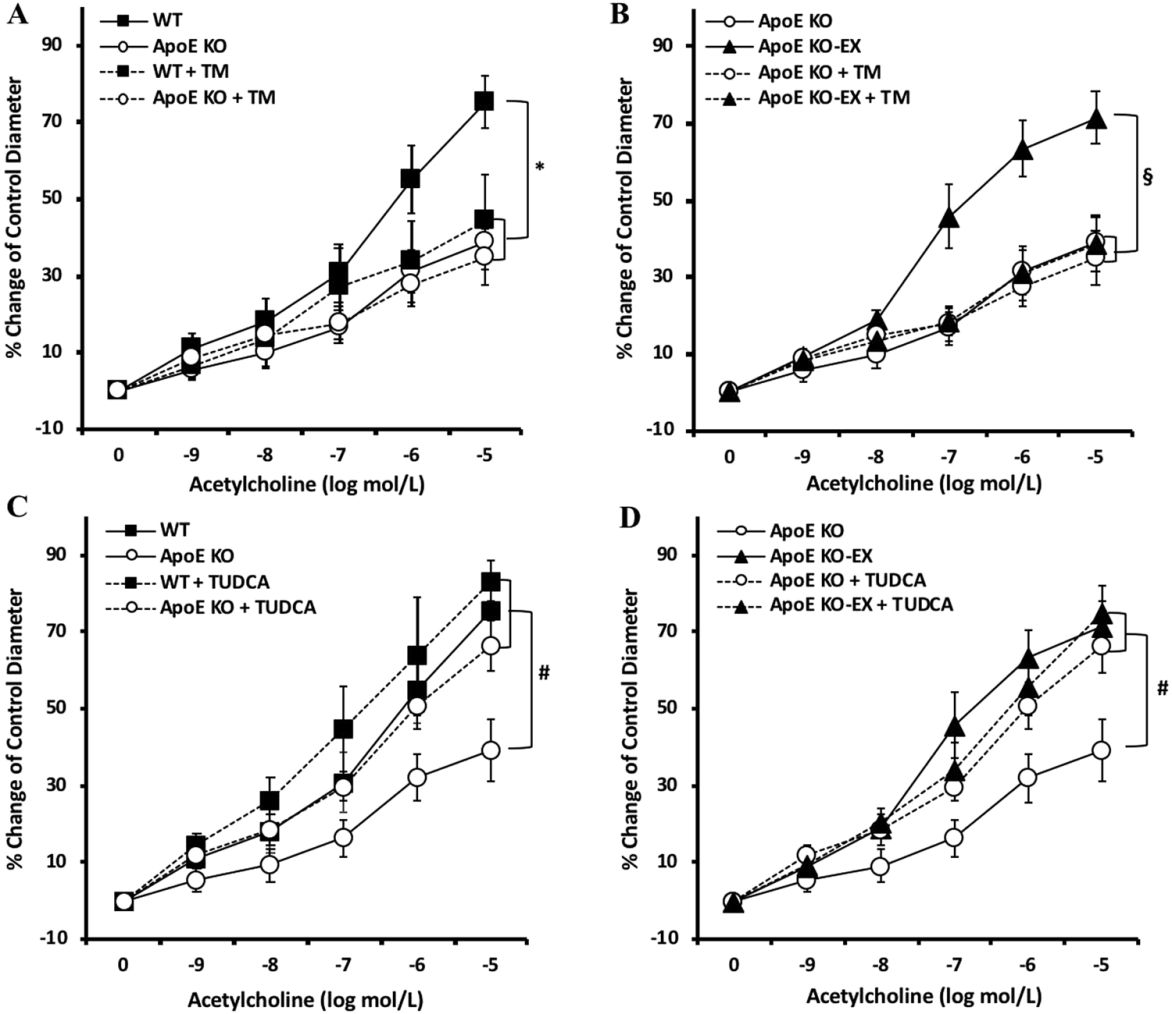
Effects of exercise training on ER stress-mediated endothelial dysfunction in mesenteric arteries of ApoE KO mice. (**A** and **B**) Incubation of the ER stress inducer, Tunicamycin (TM), in the isolated mesenteric arteries significantly attenuated endothelial-dependent vasodilation in WT + TM (n = 7) and ApoE KO-EX + TM (n = 7). (**C** and **D**) Incubation of the ER stress inhibitor, Tudca, in the isolated mesenteric arteries and ACh-induced vasodilation were significantly augmented in ApoE KO + Tudca (n = 9). Values are means ± SEM. ^*^p < 0.05 vs. WT, ^#^*p* < 0.05 vs. ApoE KO, ^§^*p* < 0.05 vs. ApoE KO-EX.

To determine the contribution of ER stress to the vascular dysfunction in mesenteric arteries of ApoE KO mice, ACh-induced vasodilation was measured following treatment with an ER stress inducer (Tunicamycin) and an ER stress inhibitor (Tudca). ACh-induced vasodilation with incubation of Tunicamycin was significantly attenuated in WT indicating that ER stress can directly impair endothelium-dependent vasodilation (Fig. 3A) but tunicamycin had no additional effect on ACh-induced vasodilation in ApoE KO mice. In contrast, exercise treatment restored ACh-induced vasodilation in ApoE KO and this restored vasodilatory function was abolished by Tunicamycin treatment (Fig. 3B). Furthermore, treatment of Tudca had little improvement of ACh-induced vasodilation in WT, but it was significantly enhanced ACh-induced vasodilation in ApoE KO (*p* < 0.05) to a comparable extent as exercise in ApoE KO-EX (Fig. 3C, D). These findings directly suggest that ER stress is acutely causative of endothelial dysfunction in the mesenteric arteries of ApoE KO mice. Importantly, exercise training appears to reverse the ER stress-mediated endothelial dysfunction in these arteries.

**Figure 4:**
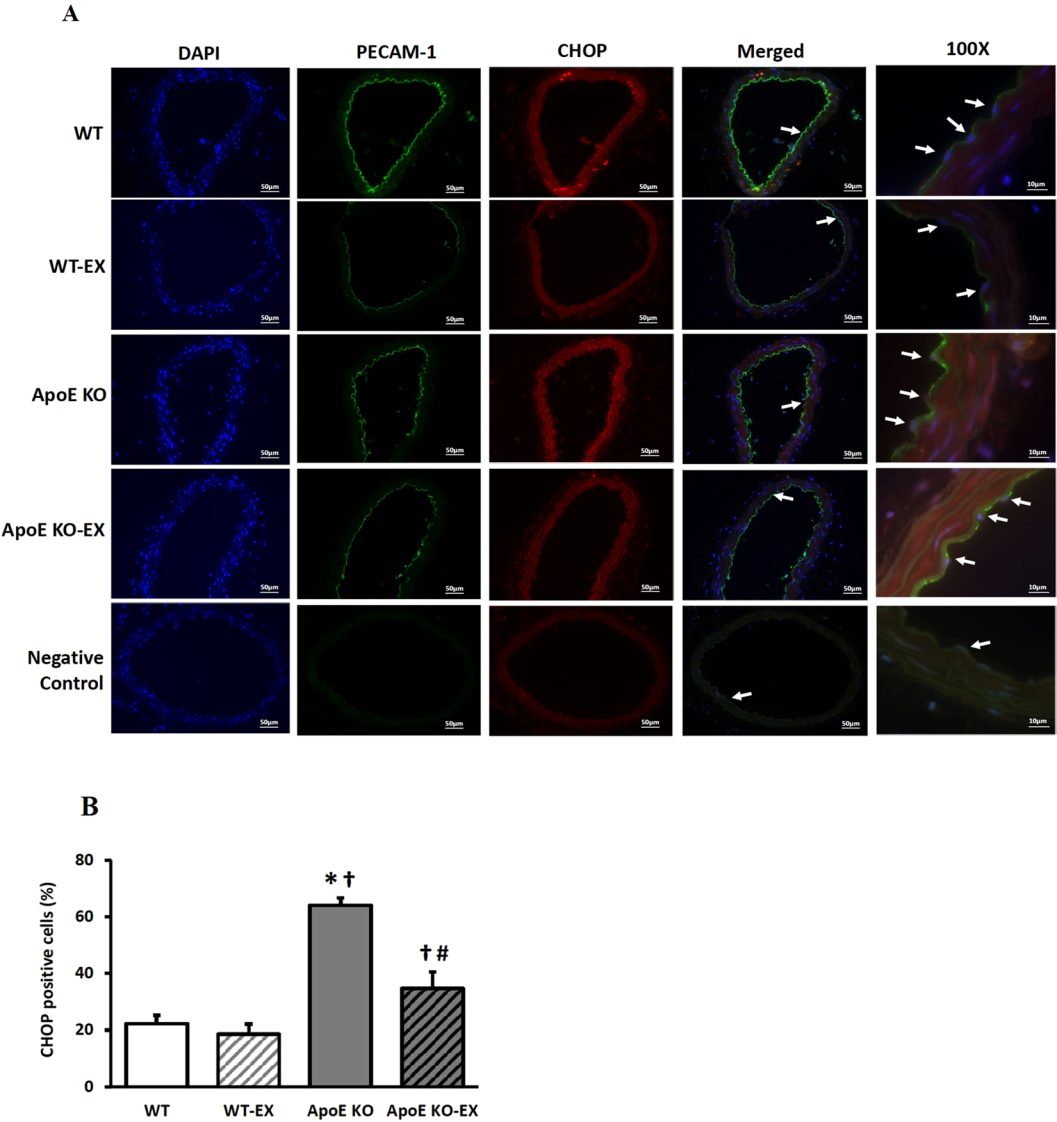
Expression and localization of CHOP with endothelial cells in the isolated superior mesenteric arteries of ApoE KO mice. (**A**) Immunofluorescence of DAPI (blue) CHOP (red), and PECAM-1 (green) in WT, WT-EX, ApoE KO, and ApoE KO-EX in the isolated superior mesenteric arteries. CHOP is colocalized with endothelial cells in the isolated superior mesenteric arteries. a, f, k, and p; dual staining of CHOP (red) and endothelial cells (green) in control mice. b, g, l, and q; dual staining of CHOP (red) and endothelial cells (green) in WT EX mice. c, h, m, and r; dual staining of CHOP (red) and endothelial cells (green) in ApoE KO mice. d, i, n, and s; dual staining of CHOP (red) and endothelial cells (green) in ApoE KO-EX mice. j and o; negative control with an absence of staining with primary antibodies. t, negative control with absence of staining with primary antibodies. Arrows show colocalization of CHOP in endothelial cells. (**B**) CHOP positive-stained mesenteric arteries were counted per section (n = 3); magnification is X 20 (scale bar; 50 µm) and X 100 (scale bar; 10 µm). ^*^p < 0.05 vs. WT, ^†^*p* < 0.05 vs. WT-EX, ^#^*p* < 0.05 vs. ApoE KO, ^§^*p* < 0.05 vs. ApoE KO-EX.

Immunofluorescence analysis showed that ER stress marker, CHOP (red, Fig. 4A k–n), was expressed in the vascular wall and co-localized with the vascular endothelial cell (green, Fig. 4A f–i) of mesenteric arteries. The expression of CHOP was significantly elevated in the vascular wall and in the mesenteric endothelial cells of ApoE KO (Fig. 4A m) compared with WT and WT-EX (Fig. 4A k,l and B), but was significantly decreased in ApoE KO-EX (Fig. 4A n,B). These results demonstrate that ER stress is highly expressed in the endothelium and is associated with endothelial dysfunction, and that exercise training ameliorates ER stress-mediated endothelial dysfunction via the diminished expression of CHOP in the endothelial cells of mesenteric arteries of ApoE KO mice.

### Exercise training ameliorates oxidative stress and inflammation in mesenteric arteries of ApoE KO

**Figure 5:**
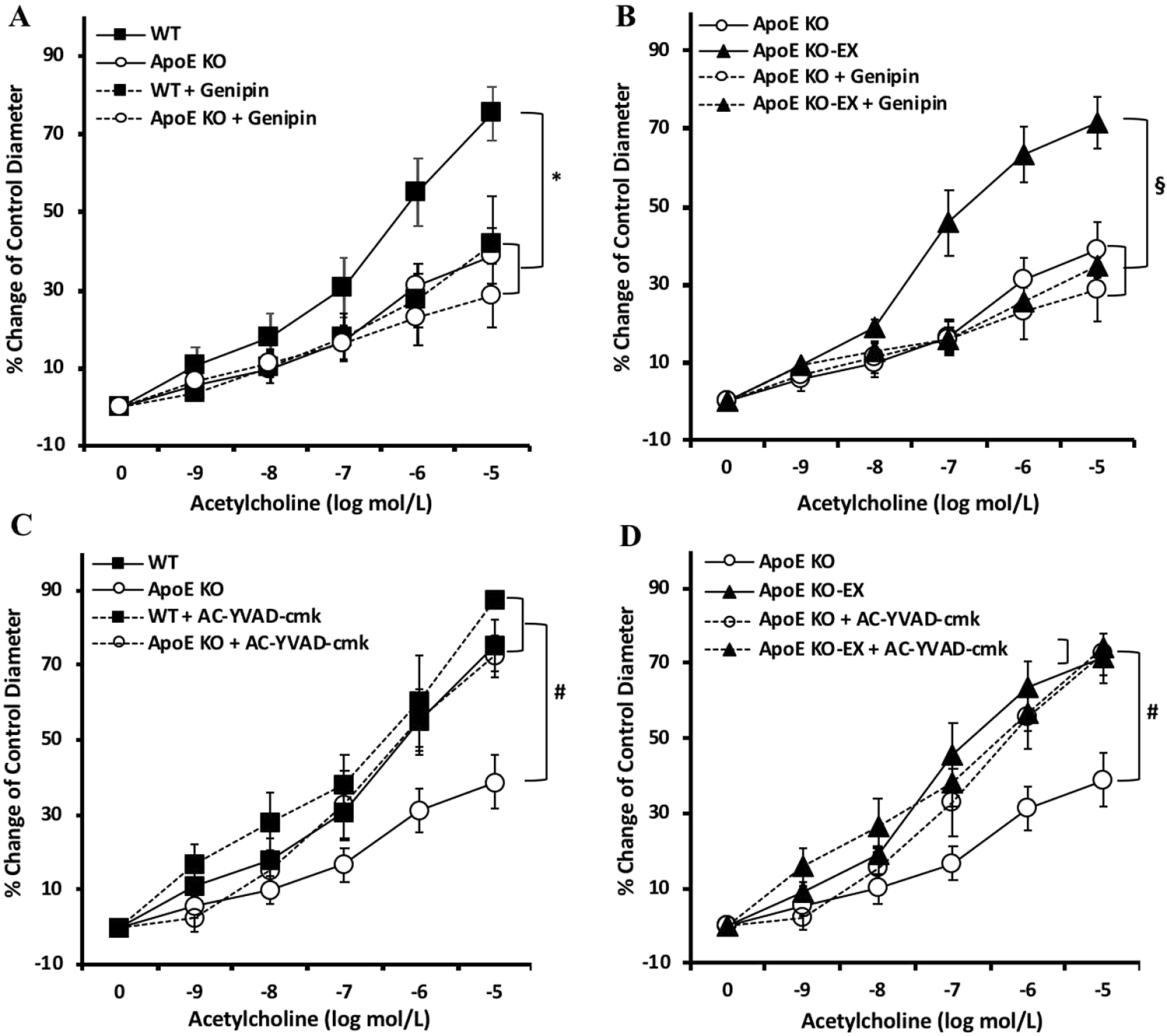
Role of caspase-1 and UCP-2 in endothelial dysfunction in mesenteric arteries of ApoE KO mice. (**A** and **B**) Incubation of the UCP-2 inhibitor, genipin, in isolated mesenteric arteries diminished ACh-induced vasodilation in WT + genipin (n = 7) and ApoE KO-EX + genipin (n = 7). (**C** and **D**) Anti-caspase-1 treatment in the isolated mesenteric arteries improved endothelium-dependent vasodilation in ApoE KO + AC-YVAD-cmk (n = 7). Values are means ± SEM. ^*^p < 0.05 vs. WT, ^#^*p* < 0.05 vs. ApoE KO, ^§^*p* < 0.05 vs. ApoE KO-EX.

To establish the role of oxidative stress in endothelial dysfunction in atherosclerotic mesenteric arteries, arteries of all groups were incubated with, genipin, a UCP-2 inhibitor. In the presence of genipin, ACh-induced vasodilation was significantly attenuated in WT and ApoE KO-EX (*p* < 0.05), but not reduced further in ApoE KO (Fig. 5A). However, genipin did effectively abolish the restored ACh-induced vasodilation in the the ApoE KO-EX mice (Fig. 5B). These results suggest that ER stress-associated oxidative stress in atherosclerotic mesenteric arteries impairs endothelial-dependent vasodilation, and that exercise training ameliorates endothelial dysfunction in the mesenteric arteries of ApoE KO mice.

To determine the contribution of caspase-1-mediated inflammation to endothelial dysfunction in mesenteric arteries of ApoE KO mice, arteries of all groups were incubated with, caspase-1 inhibitor, AC-YVAD-cmk. Treatment of AC-YVAD-cmk in the mesenteric arteries of ApoE KO mice significantly restored ACh-induced vasodilation (*p* < 0.05) which was comparable to WT and ApoE KO-EX (Fig. 5C, D). This result demonstrates that caspase-1 is involved in the mechanism of endothelial dysfunction in mesenteric arteries of ApoE KO mice, and that exercise alleviates caspase-1-associated endothelial dysfunction in ApoE KO mice.

## Discussion

The present findings show the contribution of ER stress to the endothelial dysfunction and the effects of exercise training to reverse ER stress-mediated vascular dysfunction in mesenteric arteries of ApoE KO mice. The key findings are as follows: 1) Exercise training normalized heart/body weight ratio and atherosclerotic region in ApoE KO mice; 2) ACh-induced endothelium-dependent vasodilation was impaired in mesenteric arteries of ApoE KO mice and was ameliorated by exercise training through restored endothelial NO signaling; and 3) The changes mediated by ER stress were associated with caspase-1 and UCP-2. Taken as a whole, the results of this study suggest that exercise training acts as a significant moderator of endothelial dysfunction by attenuating ER stress and ER stress-associated inflammation and oxidative stress in mesenteric arteries of ApoE KO mice.

Atherosclerosis is associated with pathological cardiac hypertrophy by increased aortic stiffness and prolonged hypertension^19^, but exercise training could normalize this pathological cardiac hypertrophy as evidenced by reduced LV wall thickness, cross sectional area of cardiomyocyte, and heart/body weight ratio^20,21^. In accordance with previous findings, these new findings reinforce that heart weight and heart/body weight ratio significantly increased in WD fed ApoE KO mice compared to other groups (Table 1), and that exercise training significantly decreased heart weight and heart/body weight ratio in ApoE KO, which was comparable to the other groups. This finding provides direct evidence of the effectiveness of exercise as a therapeutic intervention (Table 1), and demonstrates that exercise training may normalize pathological cardiac hypertrophy in ApoE KO mice. Furthermore, exercise training significantly reduced the elevated atherosclerotic lesion in the aorta of WD fed ApoE KO (Fig. 1A,B). Previous studies reported that swimming and aerobic exercise training markedly alleviated atherosclerosis in the diminished atherosclerotic lesions area, fatty streak formation, and fibrofatty plaques of aorta in the atherosclerosis mouse models^22,23^.

NO, a potent vasodilator released from endothelial cells, is associated with the mechanism of atherosclerosis initiation and progression. Endothelial dysfunction is an initial parameter of atherosclerosis and the impaired endothelial function of resistance vessels in the ApoE KO mice is characterized by reduced NO bioavailability^24^. In this study, ACh-induced vasodilation was impaired in the mesenteric arteries of ApoE KO (Fig. 2A), but no difference was observed in SNP-induced endothelium-independent vasodilation (Fig. 2B). Furthermore, incubation with L-NAME did not alter endothelium-dependent vasodilation in ApoE KO, while it drastically reduced it in the other groups (Fig. 2C, D). These findings suggest that atherosclerosis impairs the vascular function of mesenteric arteries in an endothelium-dependent and NO-mediated manner.

Although the effectiveness of exercise on atherosclerosis-associated vascular dysfunction has been widely studied on conduit vessel, to our knowledge the present study is the first to demonstrate that exercise training restores NO-mediated endothelial dysfunction in mesenteric arteries, which are resistance arteries. Notably, exercise training restored ACh-induced vasodilation in the mesenteric arteries of ApoE KO mice (Fig. 2A), whereas no difference was found in SNP-induced vasodilation (Fig. 2B). Moreover, L-NAME treatment in the isolated mesenteric arteries of ApoE KO-EX drastically reduced ACh-induced vasodilation, but produced no further reduction in the ApoE KO mice (Fig. 2C, D). Our work aligns with two earlier findings: swimming exercise training, which positively regulated ACh-induced endothelial dysfunction in the aorta of ApoE KO^25^, and treadmill exercise, which prevented endothelial dysfunction with the increased expression of p-eNOS, NO production, and antioxidant protein in the thoracic aorta of LDL-deficient mice^15^. Accordingly, the reduction of NO production in mesenteric arteries of ApoE KO mice in our study may also be through decreased production and/or activity of eNOS, with reversal by exercise training.

The inhibition of ER stress restored endothelial dysfunction with the increase in p-eNOS, and NO production in aorta and mesentery arteries of the hypertensive^26^ and diabetic mice^16^. The present study found that ACh-induced endothelial dysfunction in ApoE KO mice was ameliorated by Tudca, the ER stress inhibitor (Fig. 3C), and Tunicamycin, the ER stress inducer, directly impaired endothelium-dependent vasodilation in the WT and ApoE KO-EX mice, but not in ApoE KO mice (Fig. 3A, B). It reinforces that ER stress tightly correlates with endothelial dysfunction in the mesenteric arteries of ApoE KO. Furthermore, exercise training has been accepted as an effective treatment to reduce ER stress, but more studies are needed to confirm the beneficial effects of exercise on ER stress-associated vascular dysfunction^27^. Recently, Cheang *et al*. reported that treadmill exercise ameliorated endothelial dysfunction via the PPAR-𝛿 dependent mechanism with a decrease in the expression of p-eIF2α, ATF3 & 4 and an increase in intracellular NO production in the aorta and mesentery artery of db/db mice^16^. Our studies showed that reduced ACh-induced vasodilation in ApoE KO-EX mice by Tumicamycin is similar to the ApoE KO mice, and the incubation of Tudca in the ApoE KO mice enhances the vasodilatory function to the level of ApoE KO-EX (Fig. 3). These findings suggest that exercise training may restore the impaired endothelium-dependent vasodilation in mesenteric arteries of ApoE KO mice by attenuating ER stress-mediated mechanisms.

Our immunostaining data indicated that CHOP was expressed in the vascular wall, including the endothelium of mesenteric arteries and its expression was significantly elevated in ApoE KO mice (Fig. 4A, B), which suggests that the augmented CHOP-associated ER stress in the mesenteric artery contributes to endothelial dysfunction. This result is consistent with the earlier findings that CHOP expression was augmented in smooth muscle cells of the isolated coronary arteries of hypertensive rats^7^ and in endothelium of the aorta of ApoE KO^28^, and they were associated with the CHOP-mediated apoptotic signaling, which induces vascular damage^4^. Furthermore, we showed that exercise training significantly reduced the elevated CHOP expression in the mesenteric arteries of ApoE KO mice (Fig. 4A, B), which aligns with the previous findings that regular exercise training decreased the expression of CHOP in myocardial infarction rats^29^ and CHOP-mediated apoptosis proteins, Bcl-2 & Bax in obese mice^30^. The changes of CHOP expressions in endothelial cells, which may be responsible for the impaired ACh-induced endothelial function in the ApoE KO mice and decreased protein level of CHOP in mesenteric arteries by exercise training in atherosclerotic mice, may indicate a shift from a pro-apoptotic to a pro-survival UPR signal.

A significant novel finding of the current study is that the inhibition of caspase-1 alleviates the impaired ACh-induced vasodilation in atherosclerotic mesentery arteries (Fig. 5C, D), which suggests that caspase-1 is responsible for endothelial dysfunction in atherosclerosis possibly through an ER stress-dependent manner. The cleaved caspase-1 is activated by NLRP3 inflammasome, and is a biologically active form with proteolytic actions protein substrates, including pro-inflammatory cytokine interleukin (IL)-1β^31^, which associates with endothelial dysfunction and injury^32^. Caspase-1 deficiency in ApoE KO mice alleviates atherosclerosis through the reduced atherosclerotic plaque area and plasma level of inflammation (IL-1β, IL-1α, IL-6 and TNF-α)^8,9^. Furthermore, the inhibition of caspase-1 ameliorated the impaired endothelium-dependent vasodilation via upregulation of eNOS activity in the coronary arteries of hypercholesterolemia^11^ and homocysteinemia^32^. Induction of ER stress augmented the expression of caspase-1 p20 and IL-1β, but the inhibition of ER stress reduced the caspase-1 expression in the livers of ob/ob mice^33^. Wang *et al*. showed that treadmill exercise reduced the caspase-1 expression along with a decrease in expression of NLRP3/ASC and IL-1β/18 in the hippocampus of ovariectomy-induced depression-like mice^17^. However, the direct effects of exercise on caspase-1 in the vascular function have not been clearly established. Our finding indirectly suggested that exercise training may ameliorate endothelial dysfunction possibly via caspase-1 in the mesentery arteries of ApoE KO (Fig. 5C, D), but further study is needed to confirm the clear link between caspase-1 and ER stress in the endothelial dysfunction in atherosclerosis, and the specific effects of exercise training on the caspase-1 expression in the mesentery arteries of ApoE KO mice.

The UCP-2 is the mitochondrial anion carrier protein, which is a critical factor regulating a normal range of mitochondrial ROS production, and in turn maintains NO homeostasis to protect endothelial function^34^ in atherosclerosis^14,35^. Tian *et al*. found that UCP-2 deficiency impaired endothelium-dependent vasodilation with an elevated ROS and a decrease in p-eNOS expression in the aorta and mesenteric arteries, but that UCP2 overexpression by adenoviral infection of UCP2 restored the impaired endothelium-dependent vasodilation, elevated p-eNOS, and reduced ROS^14^. Figure 5A, B concurs with a previous finding that incubation of Genipin decreases ACh-induced endothelium-dependent vasodilation in WT and ApoE KO-EX, but not in ApoE KO, which indicates that the increase in ROS by UCP-2 inhibition exacerbates endothelial dysfunction in mesenteric arteries of ApoE KO mice. Moreover, it is known that the inhibition of UCP-2 increases the expression of Bip and CHOP, and IRE1α, PERK, ATF6 with the elevation of NADPH oxidative activity in macrophages^36^ and mouse aorta endothelial cells (MAECs)^37^. It has been shown that exercise training alleviates endothelium-dependent vasodilation via an increase in UCP-2 expression, i.e., chronic exercise training ameliorated ACh-induced endothelial dysfunction with the elevated UCP-2 expression^18^. However, no study has evaluated the exercise effects on UCP-2 in vascular dysfunction in any pathology including atherosclerosis. Figure 5A shows that the Genipin eliminates the exercise effect on endothelium-dependent vasodilation in the mesentery artery of ApoE KO mice (ApoE KO-EX), which is similar to the ApoE KO mice. This finding suggests that improved endothelial function in the ApoE KO mice by exercise training occurs in a UCP-2 dependent manner in mesenteric arteries of ApoE KO mice. However, further study is required to define the protective role of exercise in UCP-2-mediated vascular dysfunction in atherosclerosis.

A limitation of the current study is the absence of cellular and molecular expressions data of ER stress markers and key factors such as caspase-1, UCP-2, and eNOS affected by atherosclerosis and exercise training. This is mainly due to the small amount of tissue available and long experimental time course. Future studies would be required to more directly test the exercise effect on specific component of ER stress-associated endothelial dysfunction in atherosclerotic mesenteric arteries. Despite the limitation, this study is novel to report how of exercise ameliorate ER stress-mediated mesenteric vascular dysfunction within mesenteric resistance vessels in atherosclerosis.

## Methods

### Animal models

Male wild type mice (5 wks of age, WT, C57BL/6) and ApoE knockout mice (6 wks of age, ApoE KO, ApoE^tm1Unc^) were purchased from the Jackson Laboratory (Bar Harbor, ME). All mice were housed in an animal facility with controlled temperature (22–23 °C) and 12 hours light/dark cycles, and allowed free access to water and chow. WT and ApoE KO mice were randomly divided into exercise training (EX) and sedentary groups. WT and WT-EX mice were fed a normal chow and ApoE KO and ApoE KO with EX mice were fed a western atherogenic diet (0.2% cholesterol, 42% Kcal from fat, TD.88137, ENVIGO) for 17 wks to accelerate atherosclerosis development in the ApoE KO model. All experiments on animals were performed in accordance with the approved animal care, relevant guidelines, and regulation of the Institutional Animal Care and Use Committee at the University of Houston (14–009).

### Exercise training protocols

Briefly, WT-EX and ApoE KO-EX mice groups were randomly assigned and acclimated to run on a motorized rodent treadmill (Columbus Instruments, Columbus, OH) prior to exercise training for 1 week. The exercise training was performed at 7–8 wks of age, and the exercise training protocol consisted of up of 1 hour of running on a rodent motor-driven treadmill at 15 m/min at a 5% grade (60~80% of VO_2_ max), 5 days/week for 15–16 wks. The daily exercise protocol consisted of warm-up (5 min), running (50 min), and cool-down (5 min). Non-exercise groups of mice were housed in the same room and under the same conditions, but without the exercise training. At 24–25 wks of age, mice were sacrificed within 24 hrs after the final training day.

### Functional assessment of isolated mesenteric arteries of ApoE KO mice

After euthanasia, the mesenteric artery bed was rapidly excised and placed in a cold dissection chamber (~4 °C) with cold physiological saline solution (PSS) containing 145.0 mM NaCl, 4.7 mM KCl, 2.0 mM CaCl_2_, 1.17 mM MgSO_4_, 1.2 mM NaH_2_PO_4_, 5.0 mM glucose, 2.0 mM pyruvate, 0.02 mM EDTA, 3.0 mM MOPS buffer, and 1% of BSA at pH 7.4. The second or third branch of mesenteric artery was carefully isolated, and the connective and fatty tissues were removed from the surrounding artery under a dissecting microscope (Nikon SMZ1000). A single isolated mesenteric artery (180–220 μm in internal diameter and 0.5–1.0 mm in length) was transferred to a Lucite chamber containing PSS, cannulated with glass micropipettes filled with PSS-albumin solution, and secured with surgical nylon sutures. The chamber was transferred to a stage of an inverted microscope (Nikon Eclipse Ti-S) and the cannulated artery was pressurized to 80cmH_2_O intraluminal pressure without altering the intraluminal flow. Temperature was maintained at 36.5 °C. Vascular diameter changes were monitored by inverted microscope with video caliper (Colorado Video, Boulder, CO, USA), as previously described by our laboratory^38^. After equilibrium (1 hr), the isolated mesenteric artery was pre-constricted with phenylephrine (PE, 1–5 μmol/L) and the vasodilation function was tested by different stimuli. If the vessel constricted less than 20% of initial diameter, it was discarded. To determine whether atherosclerosis and exercise regulated vascular function in ApoE KO mice, the dose–dependent-diameter changes were established with endothelium-dependent vasodilator, ACh (0.1 nmol/L to 10 µmol/L) and endothelium-independent vasodilator, sodium nitroprusside (SNP, 0.1 nmol/L to 10 µmol/L) in mesenteric arteries from WT, WT-EX, ApoE KO, and ApoE KO-EX. Tudca (ER stress inhibitor, 10 µmol/L), Tunicamycin (ER stress inducer, TM, 10 µmol/L), AC-YVAD-cmk (caspase-1 inhibitor, 10 µmol/L), and genipin (UCP-2 inhibitor, 10 µmol/L) were each incubated with the isolated vessel for 20–30 min before measuring ACh-induced endothelium-dependent vasodilation. The contribution of NO to vasodilation was assessed by incubating the vessels with the *N*^*G*^-nitro-L-arginine methyl ester (L-NAME; NO synthases inhibitor; 10 μmol/L, 20 min). Tudca and TM were dissolved in 0.1% DMSO and all other drugs were dissolved in PSS and then extraluminally administrated into the PSS in the chamber before measuring the vascular function.

### Atherosclerotic lesions analysis

Oil Red O staining was used measure and visualize the severity of the atherosclerotic lesion. The aorta was dissected from ascending (thoracic aorta) to descending aorta (femoral artery), and adipose and connective tissue were removed and fixed in 4% of paraformaldehyde for overnight at 4 °C. The dissected samples were placed into a tube filled with 400 μm of Sudan red Vb (5 mg/mL in 70% isopropanol) and each tube was incubated in a water bath for 40 min at 37 °C. After incubation, the samples were washed with the 70% isopropanol. Total 3–4 mice per group were utilized and one whole image per mice was taken for atherosclerotic lesions analysis. The extent of the staining area was assessed under a dissecting microscope, and the percentage of plaque per total area was measured by an image analyzer (image J software, NIH, MA).

### Immunofluorescence staining

Superior mesenteric arteries were stained by immunofluorescence to visualize and localize the ER stress maker with markers of the endothelial cells. Briefly, isolated superior mesenteric arteries were fixed in 4% paraformaldehyde solution in PBS overnight, and the fixed tissue was perfused in 30% sucrose in PBS overnight at 4 °C. Once the superior mesenteric artery was completely perfused, the tissue was embedded into tissue-tek O.C.T. compound, snap frozen at −60 °C, and stored at −80 °C. The frozen samples were sectioned at 5 µm thick using cryostat (Leica), the sections were placed on microscope slides, and the slides were dried at room temperature (RT) for 10–15 min and washed 3 times in PBS (each 10 min) to remove O.C.T compound. Heat induced epitope retrieval (sodium citrate buffer, pH 6.0) was performed on the washed slides for 20 mins, and the slides were cooled for 20 min and then washed 3 times in PBS. For cell permeabilization, the slides were incubated in 1% of triton X-100 for 15 min and washed 3 times in PBS. Blocking buffer (5% donkey serum in PBS) was then applied for 30 min at RT. Primary antibodies, diluted in blocking buffer, were incubated: mouse IgG CHOP (CST, #2895, 1:100), and goat IgG PECAM-1 (Santa Cruz, M-20, 1:400) overnight at 4 °C. After washing the slides 3 times in PBS, secondary antibodies, diluted in PBS, were incubated: FITC (Donkey anti-goat IgG, Alexa 488-conjugated) or Texas Red (donkey anti-mouse, Alexa 594-conjugated) for 2 h at RT and washed 3 times in PBS. After washing, the slides were mounted in mounting media with DAPI (ProLong® Diamond Antifade Mountant with DAPI, Thermo Fisher Scientific) with a cover slide in the darkroom. Stained tissues were observed with a fluorescence microscope (Olympus BX51) and 20X and 100X objective, imaged with a color camera (Olympus DP73), and the digital images acquired with image software (Olympus cellSens Dimension). For negative controls, the primary antibody was omitted in the protocol and samples imaged using the same parameters. For quantification, images were acquired from 3–4 mice per group with 20X objective lens, and cells were quantified using the cell counting function of the NIH Image J software. Total 3–4 mice per group were taken and one image per mice was used for immunofluorescence. Typical ranges of total cell count per tissue were 10–20 in the endothelial cell layer.

### Data analysis

All diameter changes to pharmacological agonists were normalized to the control diameter. Normalized diameters were averaged at each concentration of agonist used and shown as mean ± SEM. Statistical comparisons of vasoreactivity responses between groups were performed with two-way analysis of variance (ANOVA) for repeated measure and intergroup differences were tested with Fisher’s protected LSD test. The significance of intergroup differences observed in body weight and vessel diameters were analyzed with one-way analysis of variance (one-way ANOVA) using software SPSS 22.0. Significance was accepted at *p* < 0.05.
